# Beyond steroid monotherapy: a systematic review and meta-analysis of combination therapy for severe to profound sudden sensorineural hearing loss

**DOI:** 10.1016/j.bjorl.2026.101849

**Published:** 2026-07-30

**Authors:** Carolina Tobar Palacios, Laura Valentina Pulido González, Linda Vanessa Gómez Triana, María Victoria Barrios Olaya, Paula Daniela Sánchez Peña, Juan Pablo Alzate Granados

**Affiliations:** a Universidad Icesi, Cali, Colombia; b Universidad Militar Nueva Granada, Bogotá, Colombia; c Fundación Universitaria Juan N. Corpas, Bogotá, Colombia; d Universidad de La Sabana, Bogotá, Colombia; e Fundación Universitaria de Ciencias de la Salud (FUCS), Bogotá, Colombia; f National University of Colombia, Bogotá, Colombia

**Keywords:** Sudden hearing loss, Sensorineural hearing loss, Corticosteroids, Combination therapy, Drug therapy

## Abstract

**Objective:**

To systematically evaluate the comparative efficacy of initial combination therapy with systemic and intratympanic corticosteroids versus systemic corticosteroid monotherapy for hearing recovery in patients with severe or profound Sudden Sensorineural Hearing Loss (SSNHL).

**Methods:**

A systematic review was conducted in PubMed, EMBASE, and LILACS through August 2025. Eligible studies included adults with severe or profound idiopathic SSNHL comparing systemic corticosteroid monotherapy with systemic plus intratympanic corticosteroid therapy. The primary outcome was hearing recovery measured by Pure-Tone Average (PTA) or standardized audiometric scales. Secondary outcomes included recovery rates, speech discrimination, tinnitus, and adverse events. The risk of bias was assessed using the RoB2 tool for randomized controlled trials and the Newcastle-Ottawa Scale (NOS) for observational studies.

**Results:**

Thirteen studies (3 randomized controlled trials, 2 case-control, 4 cohort, and 4 observational series) were included. Combination therapy consistently demonstrated superior hearing outcomes compared to systemic monotherapy. Qin et al. (2025) reported greater PTA improvement (31.0 ± 10.0 Db vs. 45.0 ± 9.8 dB) and higher complete recovery rates (46.5% vs. 29.4%, p = 0.005). Jung et al. (2016) found improved speech discrimination (72.3% vs. 57.4%, p = 0.042), and Gundogan et al. (2013) observed higher overall recovery (89% vs. 61.1%, p = 0.049). Observational cohorts supported these trends, especially with early treatment initiation. However, protocol heterogeneity and moderate-to-high risk of bias limited the strength of the evidence.

**Conclusion:**

Combined systemic and intratympanic corticosteroid therapy generally yields better hearing recovery and speech discrimination outcomes than systemic monotherapy in patients with severe or profound SSNHL. The therapeutic benefit appears greatest when treatment is initiated early. Despite promising evidence, further large-scale, standardized randomized trials are required to confirm the optimal treatment protocol.

## Highlights

• Combination therapy enhances hearing recovery in severe-profound SSNHL.
• Improves pure-tone average gain and speech discrimination ability.
• Early treatment initiation leads to superior auditory outcomes.
• Evidence limited by study heterogeneity and moderate risk of bias.
• Large, randomized trials are required to confirm clinical benefit.

## Introduction

Sudden Sensorineural Hearing Loss (SSNHL) represents a significant diagnostic and therapeutic challenge within the specialty of otology, classified as a true otologic emergency.^1^ Characterized by a rapid onset of hearing loss, typically unilateral, of at least 30 decibels (dB) across three consecutive frequencies within a 72-h period, SSNHL can substantially compromise auditory communication, adversely affect psychosocial health, and reduce overall quality of life.^2^ While a subset of patients may experience spontaneous recovery, the prognosis becomes markedly poor for those presenting with severe (>70 dB loss) or profound (>90 dB loss) hearing impairment. In this subgroup, the probability of achieving meaningful hearing recovery with standard therapies remains markedly low, highlighting a critical unmet need for the development of more effective treatment strategies.^3^

For decades, systemic corticosteroids have constituted the mainstay of SSNHL management, based on their potent anti-inflammatory effects intended to mitigate suspected viral, vascular, or autoimmune mechanisms. The 2019 clinical practice guidelines from the American Academy of Otolaryngology-Head and Neck Surgery (AAO-HNS) recommend an initial course of corticosteroids for patients with SSNHL.^1^ Despite this established standard of care, the clinical efficacy of systemic monotherapy is incomplete, and a significant proportion of patients, particularly those with poor prognostic factors such as profound hearing loss, vertiginous symptoms, and delayed treatment initiation, fail to achieve functional hearing restoration.^4^^,^^5^

This limit in treatment effectiveness has led to active research on new alternative and complementary therapies to improve recovery in SSNHL, with Intratympanic (IT) corticosteroid administration emerging as a leading contender. The primary rationale for IT therapy is pharmacokinetic; direct application into the middle ear bypasses the blood-perilymph barrier, allowing corticosteroid levels in the inner ear fluids to be much higher than those reached with systemic administration, all while minimizing systemic side effects.^6^ Consequently, the combination of systemic and intratympanic therapy represents a rational strategy to optimize drug delivery through two distinct routes, with the potential for a synergistic clinical benefit.

The investigation of this hypothesis has driven a significant increase in clinical research; however, the collective evidence remains equivocal. Some studies, including high impact randomized controlled trials published in leading journals and focusing specifically on severe to profound hearing loss, report a significant benefit for primary combination therapy over systemic monotherapy.^7^ In contrast, other high-quality meta-analyses and systematic reviews have concluded that there is no statistically significant difference in hearing outcomes between the two approaches, questioning the routine use of a more invasive initial treatment.^8^ This inconsistency in the literature has resulted in clinical uncertainty, preventing physicians from having a definitive, evidence-based approach to manage the most severe cases.

Given that patients with severe to profound SSNHL face the worst prognosis, they represent the population in which a superior treatment modality is most needed. Previous reviews often include patients with all degrees of hearing loss, which can make it harder to detect the real benefit of treatment in the most affected group. Therefore, we urgently need a thorough review of the evidence that focuses only on this high-risk group. This systematic review aims to address this knowledge gap by evaluating the comparative efficacy of initial combination therapy (systemic and intratympanic corticosteroids) versus systemic corticosteroid monotherapy on hearing recovery, specifically in patients with severe or profound SSNHL. The findings are intended to clarify the existing controversy and provide robust evidence to guide clinical decision-making for this challenging patient cohort.

## Methods

This study was conducted as a systematic review of the literature. We included only primary research articles that evaluated patients with severe or profound idiopathic sudden sensorineural hearing loss and compared initial combination therapy, defined as systemic plus intratympanic corticosteroids, against systemic corticosteroid monotherapy. Case reports and case series were excluded in order to ensure the inclusion of studies with sufficient methodological rigor and sample size to allow meaningful interpretation of outcomes. Eligible participants were adults diagnosed with sudden sensorineural hearing loss greater than 70 dB, and studies that did not specify the degree of hearing loss or that included mixed populations without subgroup analysis for severe or profound cases were excluded.

The primary outcome was hearing recovery, measured by pure tone audiometry or other standardized audiometric scales, as reported by each study. Secondary outcomes included rates of complete recovery, partial recovery, and adverse effects related to therapy.

To identify relevant studies, we performed comprehensive searches in PubMed, EMBASE, and LILACS from inception to August 2025. The search strategy combined controlled vocabulary terms and free-text words related to “sudden sensorineural hearing loss”, “severe”, “profound”, “corticosteroids”, and “combination therapy”. The complete search strategies for each database were predefined and adapted to the indexing system of each source. No language or publication status restrictions were applied. In addition, reference lists of included articles and relevant reviews were screened to identify additional eligible studies.

Two reviewers independently screened the titles and abstracts retrieved from the searches and subsequently assessed the full texts of potentially relevant articles. Disagreements were resolved through discussion or consultation with a third reviewer. Data extraction was carried out independently by two reviewers using a standardized form that captured study design, participant characteristics, intervention details, outcomes, and results.

The methodological quality and risk of bias of the included studies were assessed using validated critical appraisal tools appropriate for each study design. Particular attention was given to randomization, blinding, completeness of follow-up, and selective outcome reporting. Any discrepancies in assessment were resolved by consensus. Because of the clinical and methodological heterogeneity across the included studies, a meta-analysis was not conducted. Instead, the results were synthesized narratively, with findings grouped by type of intervention and outcome. Where appropriate, similarities and differences in study design, populations, and outcome definitions were highlighted to provide context for the interpretation of the evidence.

## Results

### Search results

**Fig. 1:**
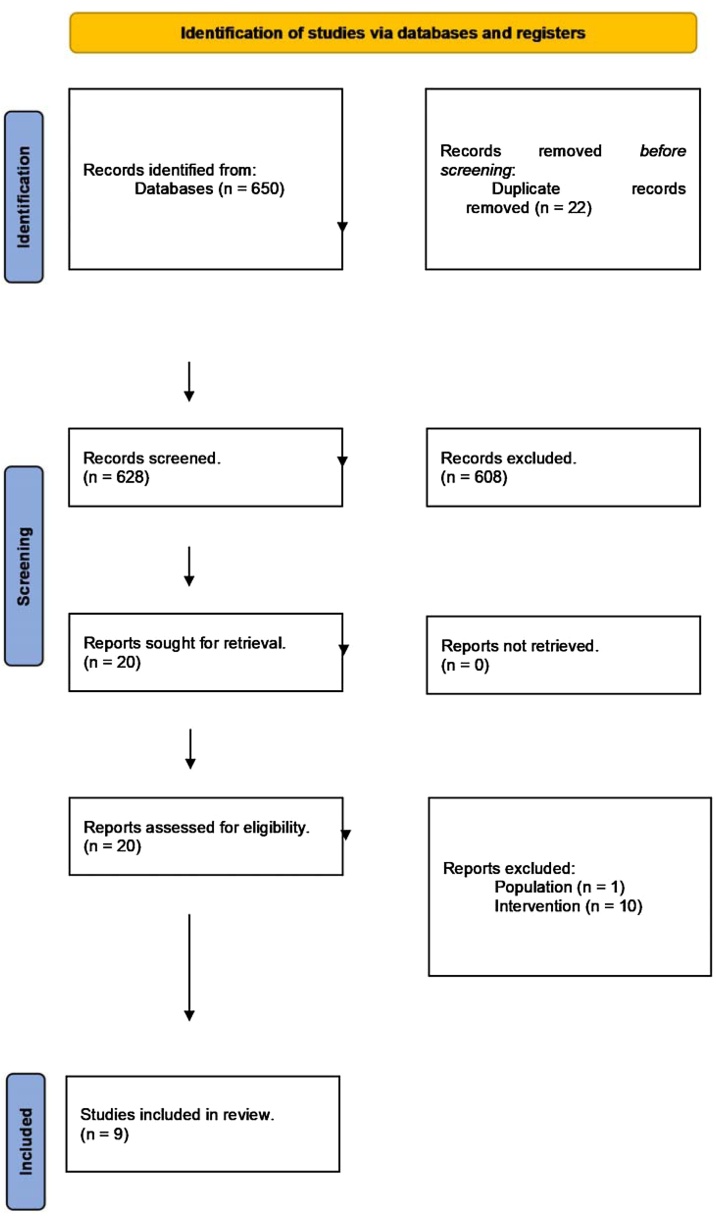
PRISMA flow diagram.

We identified 650 records from databases; after removing 22 duplicates (3.4%), 628 records underwent title/abstract screening, of which 608 (96.8%) were excluded. We sought and retrieved 20 full-text reports for eligibility assessment. Of these, 11 were excluded ‒ 1 for wrong population and 10 for inappropriate intervention (55.0% of full texts). Ultimately, 9 studies met the inclusion criteria and were included in the review (1.4% of all identified records; 45.0% of those assessed at full text) (Fig. 1).

Across the included studies, the efficacy of intratympanic and combined steroid regimens for Sudden Sensorineural Hearing Loss (SSNHL) was consistently demonstrated, although with some variability in the magnitude of the effect depending on the study design, patient characteristics, and treatment modality. In the randomized trial by Qin et al. (2025), the combination of intratympanic methylprednisolone sodium succinate with acoustic resonance therapy produced significantly greater improvements in pure-tone average (PTA), speech discrimination, and tinnitus reduction compared with intratympanic methylprednisolone alone or placebo ^9. By day-60, patients in the combination therapy group achieved a mean PTA of 31.0 ± 10.0 dB, compared to 45.0 ± 9.8 dB in the monotherapy group and 65.0 ± 12.5 dB in the placebo group ^9. Recovery rates were also highest in the combined therapy arm, where nearly half of the patients (46.5%) achieved a complete recovery ^9. These findings were paralleled by reductions in systemic inflammatory markers (CRP, IL-6, TNF-α, fibrinogen) and improvements in cerebral microcirculation, suggesting a dual therapeutic mechanism involving both local cochlear and systemic vascular-inflammatory pathways.^9^

In contrast, salvage therapy studies such as those by Liang and Li (2020) and Demirhan et al. (2018) evaluated patients who had failed initial systemic treatment.^10^^,^^11^ Here, combined systemic and intratympanic steroid therapy showed higher response rates compared with intratympanic therapy alone, though the differences did not always reach statistical significance.^10^^,^^11^ Improvements in PTA ranged from 7.5 Db to 16.3 dB, and recovery was most favorable when treatment was initiated within 30-days of symptom onset.^10^ Both tinnitus-related distress (measured by THI and VAS) and anxiety symptoms (HAMA scores) improved significantly in all groups, but without meaningful differences between the intratympanic and combined approaches.^10^^,^^11^

Evidence from Jung et al. (2016) and Gundogan et al. (2013) further supports the benefit of combined approaches, particularly among patients with severe to profound hearing loss.^12^^,^^13^ Jung et al. observed a significantly higher improvement in speech discrimination scores at 90-days in the combined group (72.3%) compared with systemic therapy alone (57.4%).^12^ Similarly, Gundogan et al. demonstrated a markedly greater rate of overall auditory recovery with combination therapy (89%) compared to oral steroids alone (61.1%), with statistically significant gains across low, mid, and high frequencies.^13^

Large retrospective series, such as that of Kim et al. (2014), confirmed these patterns at the population level, reporting recovery rates of 73.7% with combination therapy, compared with 68.7% for oral steroids alone and 59.0% for intratympanic injections. Importantly, outcomes were more favorable in patients with milder initial hearing loss, while profound cases remained less responsive despite aggressive therapy.^14^

Finally, in treatment-refractory cohorts, such as the prospective case–control study by Filipo et al. (2017), intratympanic dexamethasone via an indwelling catheter yielded clinically significant improvements in 75% of patients who had failed systemic therapy, compared with 35% of controls. This underscores the role of intratympanic therapy as a salvage strategy, particularly in advanced or non-responsive cases.^15^

Taken together, these findings indicate that combined systemic and intratympanic corticosteroid regimens generally offer superior auditory and functional outcomes compared with monotherapy, particularly when treatment is initiated early, and the baseline hearing loss is less severe. However, heterogeneity in study design, treatment protocols, and patient selection underscores the need for further standardized, prospective trials to consolidate these results. Table 1 summarizes the included studies and their main findings.

**Table 1:** Characteristics and clinical outcomes of included studies.

Study (Author, Year)	Population & Severity	Combination Therapy (CT) Protocol: Dose, Timing, and Volume	Main Outcomes (CT vs. Systemic monotherapy)
Qin et al., 2025	n = 256 (unilateral SSNHL)	Dose: IT MPSS (20 mg/mL)	Complete Recovery: 46.5% vs. 29.4% (p = 0.005)
	Severe: 57% (33)	Volume: 0.5 mL	PTA (day 60): 31.0 dB vs. 45.0 dB (p < 0.001)
	Profound: 43%	Timing: 7 injections (every 2-days)	
Demirhan et al., 2018	n = 204	Dose: IT MP (66.6 mg/mL) or DX (4 mg/mL)	Success Rate: 55% vs. 34% (p = 0.004)
	Severe: 25% (11)	Volume: 0.5‒0.7 mL	Severe Subgroup (70–89 dB): CT significantly higher (p = 0.0001)
	Profound: 48.3%	Timing: 5 sessions (every 72 -hs)	
Jung et al., 2016	n = 105	Dose: IT Dexamethasone ($5\text{mg/mL})	SDS (90 days): 72.3% vs. 57.4% (p = 0.042)
	Severe-Profound: > 70 dB	Volume: 0.3‒0.7 mL	PTA (90 days): 43.0 dB vs. 54.8 dB (p = 0.013)
		Timing: 5 injections (every 2-days)	
Gundogan et al., 2013	n = 73	Dose: IT Methylprednisolone (62.5 mg/mL)	Recovery (Siegel): 89% vs. 61.1% (p = 0.049
	Severe-Profound cases included	Volume: 0.4 mL	SDS Gain: 41.08% vs. 20.06% (p = 0.000)
		Timing: 4 injections (over 2-weeks)	
Filipo et al., 2017	n = 127 (refractory)	Dose: IT Dexamethasone (4 mg/mL)	Improvement: 75% vs. 35.4% (controls) (p < 0.001)
	Severe-Profound: 100%	Method: Infusion via 4 Fr catheter	PTA Gain: 24.0 dB vs. 6.7 dB (p < 0.001)
		Timing: Daily (max 7-days)	
Liang & Li, 2020	n = 90 (refractory)	Dose: IT Methylprednisolone (40 mg/mL)	Success Rate: 51.1% vs. 31.1% (p = 0.261)
	Moderate-Severe to Profound	Volume: 0.5‒0.7 mL	PTA Gain: 16.3 dB vs. 7.5 dB (p = 0.261)
		Timing: 4 injections (every 2-days)	
Kim et al., 2014	n = 844	Dose: IT Dexamethasone (5 mg/mL)	Final Recovery: 73.7% vs. 68.7% (Oral) vs. 59.0% (IT alone) (p = 0.031)
	Stratified (Mild to Profound)	Volume: 0.3‒0.4 mL	
		Timing: 4 injections (twice weekly)	

Across the three eligible randomized controlled trials, overall risk of bias was mostly some concerns, with one study at high risk. The Qin 2025 trial reported computer-generated randomization with blinding for intratympanic injections and blinded outcome assessment, supporting low risk for randomization and measurement, though partial unblinding of the acoustic-resonance component and lack of a pre-specified analysis plan yielded some concerns overall. The Wang 2025 study stated random assignment to four arms and used objective audiometry, but did not describe allocation concealment or attrition handling, leading to some concerns in randomization, deviations, missing data, and reporting. In contrast, the Gündoğan 2013 trial was non-blinded and analyzed per-protocol after excluding six randomized participants, which elevates risk for deviations from intended interventions and contributes to a high overall judgment despite objective outcome measurement (Table 2).

**Table 2:** Risk of bias clinical trials (RoB2).

Domain (RoB2)	Wang 2025 ‒ American Journal of Otolaryngology	Qin 2025 ‒ European Journal of Medical Research	Gündoğan 2013 ‒ Otolaryngology-Head & Neck Surgery
1. Bias arising from the randomization process	Some concerns	Low	Some concerns
2. Bias due to deviations from intended interventions (effect of assignment)	Some concerns	Some concerns	High
3. Bias due to missing outcome data	Some concerns	Some concerns	Some concerns
4. Bias in measurement of the outcome	Low	Low	Low
5. Bias in selection of the reported result	Some concerns	Some concerns	Some concerns
Overall risk of bias	Some concerns	Some concerns	High

Across the two eligible case-control studies, Selection was limited by hospital-based controls and the absence of independent case validation, yielding only one star per study. Comparability was acceptable in Jung et al. due to adjustment for the key prognostic factor (pretreatment PTA), but not in the catheter study, which lacked multivariable control despite age/sex matching. Exposure ascertainment was strong in both ‒ treatment was captured via secure clinical records and applied using uniform procedures; Jung et al. also reported complete follow-up at prespecified time points. Overall, Jung et al. was rated Moderate risk of bias (5/9 stars), whereas the catheter case-control was High risk of bias (3/9 stars), primarily due to residual confounding and control selection (Table 3).

**Table 3:** Risk of bias case-control studies (NOS).

Study (first author, year)	Selection (0–4★)	Comparability (0–2★)	Exposure (0–3★)	Overall NOS (0–9★)	Overall risk of bias
Jung et al., 2016 Audiol Neurootol	1★	1★	3★	5★	Moderate
(Prospective) catheter IT-DEX study, 2014–2016 (University of Brescia)	1★	0★	2★	3★	High

In four observational cohorts, selection was generally adequate (hospital cohorts with clear exposure ascertainment) and outcomes were measured objectively with standardized audiometry and predefined recovery criteria; follow-up ranged from ∼2–12 weeks, reaching ≥3-months in two studies. However, most analyses relied on unadjusted or minimally adjusted comparisons (often stratified only by baseline hearing severity or timing), which limits comparability and leaves meaningful residual confounding ‒ particularly for treatment allocation and time-to-therapy differences. Consequently, we rated all cohorts as having a moderate overall risk of bias, driven mainly by confounding risk rather than flaws in outcome assessment or follow-up (Table 4).

**Table 4:** Risk of bias cohort studies (NOS).

Study (year)	Selection (max ★★★★)	Comparability (max ★★)	Outcome (max ★★★)	Overall risk of bias
Demirhan et al., 2018, Acta Oto-Laryngologica	★★★★	★	★★	Moderate
Kim et al., 2014, Clinical Otolaryngology	★★★★	★	★★★	Moderate
Min et al., 2011, Korean J Audiol	★★★★	★	★★★	Moderate

## Discussion

This systematic review synthesized the available evidence comparing primary combination therapy with systemic corticosteroid monotherapy for patients with severe to profound Sudden Sensorineural Hearing Loss (SSNHL). The principal finding is that combined systemic and intratympanic corticosteroids appear to offer superior hearing recovery compared to systemic therapy alone. This trend was consistently observed across studies in terms of Pure Tone Average (PTA) gain and speech discrimination scores, suggesting a synergistic benefit when targeting the inner ear via two distinct routes.

Our findings align with previous meta-analyses, such as that by Gao and Liu, which support aggressive initial treatment for profound hearing loss.^16^ Unlike older reviews that included milder cases, potentially diluting the treatment effect, our focus on the severe-to-profound subgroup highlights the specific advantage of combination therapy in this high-risk population. The rationale remains pharmacokinetically sound: dual delivery maximizes cochlear drug concentration, potentially overcoming the blood perilymph barrier a mechanism particularly crucial when cochlear microcirculation is compromised.

Despite the promising trend, the limitations of the primary studies warrant careful consideration. The risk of bias assessment revealed methodological weaknesses, including lack of blinding and inadequate allocation concealment in observational cohorts. This introduces potential selection bias, where clinicians might prioritize aggressive therapy for patients with a perceived worse prognosis. Furthermore, the significant heterogeneity in corticosteroid type, dosage, and administration schedules across studies precludes the definition of a single optimal protocol, a persistent challenge in the field.^17^

From a clinical perspective, primary combination therapy represents a reasonable strategy for severe or profound SSNHL. Given the poor prognosis of this condition and the minimal systemic side effects of intratympanic injections, the potential for improved hearing likely outweighs the procedural risks. This approach supports shifting intratympanic steroids from a salvage option to a first-line adjuvant therapy. However, this decision must be guided by shared decision-making, considering patient preferences and prognostic factors such as vertigo or time to treatment.^18^

Future research requires large-scale, multicenter, randomized controlled trials to definitively establish superiority. These trials should utilize standardized protocols and uniform outcome reporting (including quality of life metrics) to allow for robust subgroup analyses. Until such high-quality evidence is available, clinicians should navigate the existing uncertainty by integrating the current best evidence with clinical judgment.

## Conclusion

The available evidence suggests that primary combination therapy with systemic and intratympanic corticosteroids is superior to systemic monotherapy for improving hearing outcomes in patients with severe to profound SSNHL. This approach leads to greater gains in pure-tone average and speech discrimination, particularly when treatment is initiated early. Despite these promising findings, the evidence is limited by methodological heterogeneity and a moderate-to-high risk of bias in existing studies. Large-scale, methodologically rigorous randomized controlled trials are urgently needed to establish a definitive, evidence-based standard of care for this challenging patient population.

## ORCID ID

Linda Vanessa Gómez Triana: 0009-0002-0772-491X

María Victoria Barrios Olaya: 0009-0005-1376-1532

Juan Pablo Alzate Granados: 0000-0001-8344-494X

## Funding

No external funding was received for this study.

## Data availability statement

The authors declare that all data are available in repository.

## Declaration of competing interest

The authors declare no conflicts of interest.
